# Metastatic diffuse follicular variant papillary thyroid cancer without cervical lymph node metastasis presenting with symptoms related to hypopituitarism

**DOI:** 10.31744/einstein_journal/2023RC0229

**Published:** 2023-07-06

**Authors:** Muhammed Kızılgül, Ömer Bayır, Bekir Uçan, Pınar Akhanlı, Hakan Düğer, Muhammed Erkam Sencar, Hayri Kertmen, Demet Yılmazer, Doğan Yazılıtaş, Güleser Saylam, Mehmet Hakan Korkmaz, Erman Çakal

**Affiliations:** 1 Department of Endocrinology and Metabolism University of Health Sciences Dışkapı Yıldırım Beyazıt Training and Research Hospital Ankara Turkey Department of Endocrinology and Metabolism, University of Health Sciences, Dışkapı Yıldırım Beyazıt Training and Research Hospital, Ankara, Turkey.; 2 Department of Otorhinolaryngology and Head & Neck Surgery University of Health Science Dışkapı Yıldırım Beyazıt Training and Research Hospital Ankara Turkey Department of Otorhinolaryngology and Head & Neck Surgery, University of Health Science, Dışkapı Yıldırım Beyazıt Training and Research Hospital, Ankara, Turkey.; 3 Department of Neurosurgery University of Health Sciences Dışkapı Yıldırım Beyazıt Training and Research Hospital Ankara Turkey Department of Neurosurgery, University of Health Sciences, Dışkapı Yıldırım Beyazıt Training and Research Hospital, Ankara, Turkey.; 4 Department of Pathology University of Health Sciences Dışkapı Yıldırım Beyazıt Training and Research Hospital Ankara Turkey Department of Pathology, University of Health Sciences, Dışkapı Yıldırım Beyazıt Training and Research Hospital, Ankara, Turkey.; 5 Department of Medical Oncology University of Health Sciences Dışkapı Yıldırım Beyazıt iskapi Yildirim Beyazit Training and Research Hospital Ankara Turkey Department of Medical Oncology, University of Health Sciences, Dışkapı Yıldırım Beyazıt iskapi Yildirim Beyazit Training and Research Hospital, Ankara, Turkey.

**Keywords:** Pituitary neoplasms, Thyroid neoplasms, Thyroid cancer, papillary, Hypopituitarism, Neoplasm metastasis, Lymph nodes

## Abstract

In this article, we present a case of diffuse follicular variant papillary thyroid carcinoma with pituitary metastasis, which is a rare cause of pituitary metastasis. The follicular variant of papillary thyroid carcinoma is an uncommon variant of papillary carcinoma. A 74-year-old male was presented with weakness, fatigue, and a decreased appetite. The patient was diagnosed with secondary adrenal and thyroid insufficiencies. Imaging revealed a pituitary mass with suprasellar extension, right cavernous sinus invasion, and optic chiasm compression. Thyroid ultrasonography revealed a nodule with a maximum size of 7.2cm in the right lobe. Cytological examination via fine-needle aspiration suggested papillary thyroid cancer. Total thyroidectomy with central and right lateral neck dissection confirmed the diagnosis of diffuse follicular variant of papillary thyroid carcinoma. Owing to visual field defects, the patient underwent transsphenoidal surgery. Histological and immunohistochemical evaluations confirmed pituitary metastasis from the papillary thyroid cancer. Radioactive iodine treatment and gamma knife radiotherapy of the pituitary gland were performed. The initiation of sorafenib treatment was deemed appropriate during the follow-up. A significant decrease in the thyroglobulin levels was observed after sorafenib treatment. Pituitary metastasis should be considered in patients diagnosed with hypopituitarism and pituitary lesions at initial evaluation. The presence of visual field defects may be an indication for neurosurgical intervention and guide both diagnosis and treatment. The management of papillary thyroid cancer and the role of treatment modalities in prognosis depend on the biological behavior of the tumor. Early diagnosis and multidisciplinary management are crucial for the treatment of these patients.

## INTRODUCTION

Papillary thyroid cancer (PTC) accounts for approximately 90% of all differentiated thyroid cancers (DTC).^(1)^ Approximately 12–64% of patients have lymph node metastases, of which approximately 10% have extrathyroidal invasion, and distant metastases are determined in 3-6% of patients.^(2)^ The follicular variant papillary thyroid carcinoma (FVPTC) is the second most common subtype of PTC and is characterized by a follicular growth pattern and typical nuclear features, such as nuclear clearing, grooves, and pseudo inclusions. Diffuse FVPTC has a considerably higher risk of local, nodal, and vascular metastases than the other FVPTC subtypes.^(3)^ Pituitary metastases (PM) are very rare, accounting for only 1% of pituitary masses, and are usually derived from the breast, lung, and gastrointestinal tract. Moreover, DTC represents only 2% of PM.^(4,5)^ Symptoms are present in approximately 7% of patients with PM.^(6)^ Patients with PM often present with diabetes insipidus; however, recent reports have demonstrated an increasing prevalence of other symptoms, particularly anterior pituitary dysfunction.^(7)^ Despite many advances in imaging modalities, differentiating PM from adenomas remains challenging.^(8)^ Early diagnosis is vital, and an approach by a multidisciplinary team (neurosurgery, radioiodine, external radiotherapy, and radiosurgery) is advised; even though they cannot provide a survival benefit, it may allow a better quality of life to be obtained.^(8,9)^ The case of a 74-year-old male who presented with symptoms related to hypopituitarism caused by metastatic diffuse FVPTC of the thyroid involving the pituitary glands.

## CASE PRESENTATION

**Figure 1:**
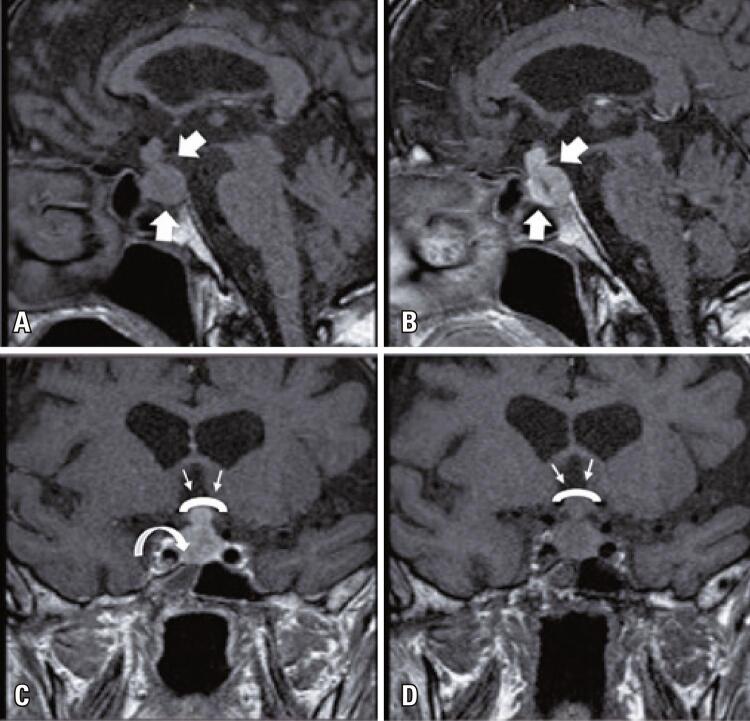
Pituitary magnetic resonance images. (A) precontrast; (B) Postcontrast sagittal T1WI magnetic resonance imaging; (C) Postcontrast; (D) Precontrast coronal T1WI magnetic resonance imaging showing pituitary mass with suprasellar extension (thick white arrows), invasion of the right cavernous sinus (curved white arrow), and optic chiasm compression (short white arrows)

**Figure 2:**
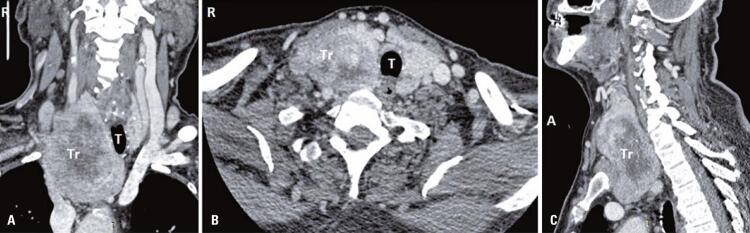
Patient’s coronal (A), axial (B), and sagittal (C) neck computed tomography images with contrast. Deviated trachea, obliterated internal jugulary vein and substernal extention of the thyroid can be observed

**Figure 3:**
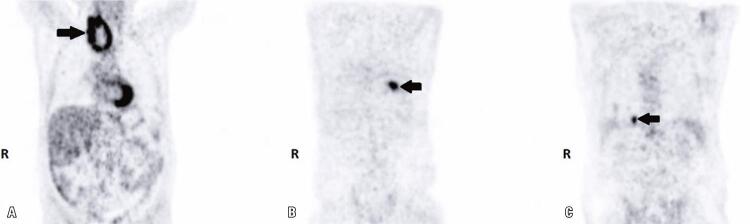
Positron emission tomographic images of the patient. Increased fluoro-deoxyglucose uptake in the thyroid gland with substernal extension (A); left 9th rib (B) and one of the pulmonary metastatic nodules (C) are seen

**Figure 4:**
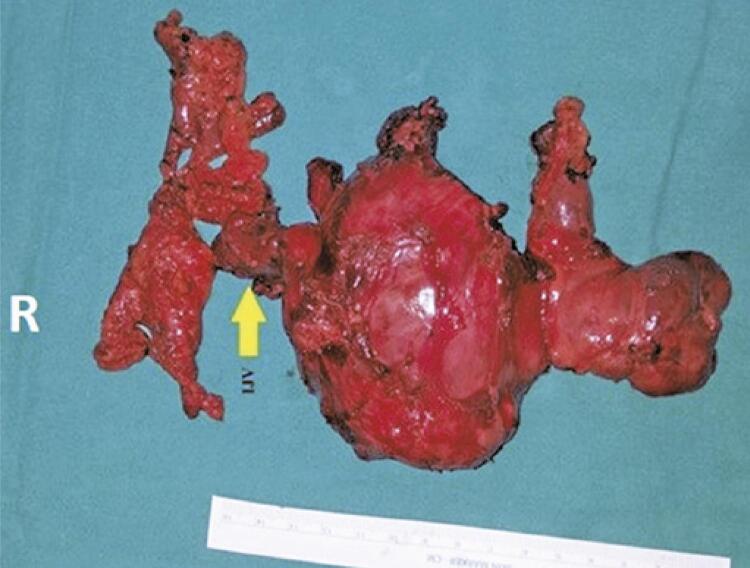
Figure showing the patient’s total thyroidectomy with central and right lateral neck lymph node dissection. The thyroid capsule was not observed over the right thyroid lobe; the tumor was extrathyroidal, and the right internal jugular vein was obliterated

**Figure 5:**
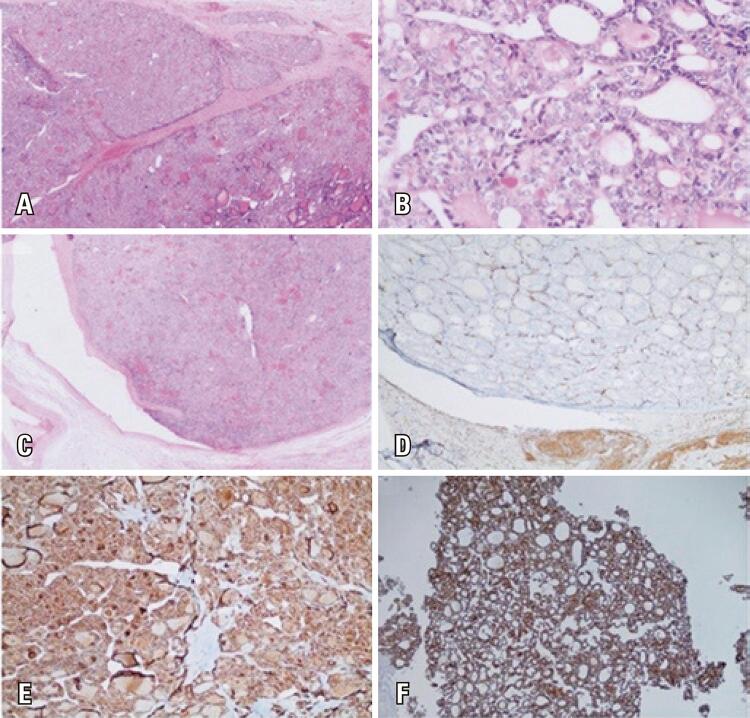
A) Histological sample of the papillary thyroid carcinoma in a multinodular developmental pattern (HE×40); B) Nuclear features of papillary carcinoma characterized by nuclear enlargement, nuclear membrane irregularity, and chromatin opening in thyrocytes with follicular array (H&E×400); C) Jugular vein obliterated by the papillary thyroid tumor (HE×200); D) CD31 (marker of endothelial differentiation) immunolabeling image of venous invasion; E) HBME-1stained papillary thyroid cancer (HBME-1×200); F) Histological sample of the pituitary tissue metastasis of the papillary thyroid cancer (Thyroglobulin ×40)

A 74-year-old male was presented with weakness, fatigue, and decreased appetite for 1 month, without an increase in urinary frequency. The patient was referred to an endocrinology outpatient clinic after the detection of low thyroid-stimulating hormone (TSH) and free thyroxine (FT4) levels during laboratory analysis. The patient’s medical history was unremarkable. Further evaluation showed decreased 8:00 a.m. cortisol, adrenocorticotropic hormone (ACTH), luteinizing hormone (LH), and follicle-stimulating hormone (FSH), and mildly elevated prolactin levels (Table 1). Hydrocortisone and levothyroxine treatments were initiated. The patient had not exhibit polyuria or polydipsia. Pituitary magnetic resonance imaging (MRI) revealed a pituitary mass with suprasellar extension, invasion of the right cavernous sinus, and compression of the optic chiasm (Figure 1). Bitemporal hemianopsia was determined by visual field examination. Pituitary surgery was considered after a consultation with a neurosurgeon. Physical examination of the thyroid gland revealed a palpable, right-sided nodule. Thyroid ultrasonography confirmed multiple nodules in both lobes and a dominant hypoechoic nodule with 7.2cm a maximum size in the right lobe. A thyroid fine needle aspiration biopsy was performed, and the result was categorized as a Bethesda category VI malignancy. During the preoperative evaluation, thoracic and neck computed tomography (CT) was performed for the substernal goiter (Figure 2). The thorax CT revealed pulmonary nodules consistent with metastases. Positron emission tomography-computed tomography (PET/CT) revealed an increased fluoro-deoxyglucose (FDG) uptake in the thyroid gland (SUVmax: 9.56), pituitary gland (SUVmax: 8.28), pulmonary nodules (SUVmax: 9.50), left 9^th^ rib (SUVmax:7.05), right-sided area adjacent to the thyroid gland, retrosternal and retroclavicular lymph nodes, and right femur (Figure 3). Biopsy results of the pulmonary nodule were consistent with thyroid carcinoma metastasis. A total thyroidectomy with central and right lateral neck lymph node dissection was performed (Figure 4). The thyroid capsule was not observed over the right thyroid lobe, the tumor was extrathyroidal, and the right internal jugular vein was obliterated (the internal jugular vein was ligated). Histopathological examination revealed diffuse FVPTC (10 × 8 × 5cm in diameter, tumor invading the adjacent tissue) in the right lobe, six foci of papillary microcarcinoma in the left lobe, tumor thrombosis in the right internal jugular vein, and no lymph node metastasis on the right side of the neck (Figure 5). Nuclear features of papillary carcinoma characterized by nuclear enlargement, nuclear membrane irregularity, and chromatin opening in thyrocytes with follicular array were observed. A transsphenoidal resection of the pituitary tumor was performed. The histopathological and immunohistochemical results of both total thyroidectomy and PM are shown in figure 5. After 1 month, the metastatic mass on the 9^th^ rib was excised with safe surgical margins, and histopathological examination revealed metastatic thyroid carcinoma. Genomic analysis revealed that the tumor did not contain any common mutations related to thyroid cancer.

**Table 1:** Baseline biochemical results of the patient

	Results	Reference range
Thyroid stimulating hormone (mIU/L)	0.11	0.38–5.33
Free T4 (ng/dL)	0.49	0.58–1.6
Free T3 (ng/dL)	4.07	2.66–4.37
Luteinizing hormone (IU/L)	0.27	1.24–8.62
Follicle stimulating hormone (IU/L)	0.85	1.27–19.26
Somatomedin C (ng/mL)	46.7	64–188
Adrenocorticotropic hormone (pg/mL)	11.6	0–46
Prolactin (ng/mL)	35.29	2.64–13.13
Cortisol (µg/dL)	1.42	6.7–22.6
Total testosterone (ng/dL)	0.1	175–781

A: anterior; R: right side; Tr: thyroid gland; T: trachea.

R: right side.

R: right side; IJV: internal jugulary vein.

Subsequently, the patient was treated with 250 mCi iodine-131 and recombinant human thyroid-stimulating hormone (rhTSH). Serum levels of thyroglobulin (Tg), Tg antibodies (anti-Tg), and TSH were measured on day 1 before the rst rhTSH injection and on day 3. The TSH and TSH-stimulated Tg levels of the patient were 147.79mLU/L and 2285ng/mL, respectively. A post-therapy whole-body scan (WBS) performed on day six revealed intense ^iodine-131^ uptake in the sphenoid bone, bilateral basal lung areas, left 12^th^ rib, right 4^th^ lumbar paraspinal area, and right kidney. The basal Tg level decreased from 1156ng/mL to 862ng/mL 1 month after radioiodine (RAI) treatment. Postoperatively, MRI of the pituitary gland indicated residual disease. The Tg, anti-Tg, and TSH levels of the patients under treatment are shown in table 2.

**Table 2:** Hormonal parameters after surgery

	TSH (mLU/L)	fT4 (ng/dL)	Thyroglobulin (ng/mL)	Anti- Thyroglobulin (IU/mL)	RAI treatment (mCİ)	Sorafenib treatment
1^st^ month	0,02	1.32	1156	237		
2^nd^ month	147.79	-	2285	-	250	
3^rd^ month	0.07	1.11	697	15		
6^th^ month	0.02	1.19	862	15		
12^th^ month	0.01	118	1960	18		
17^th^ month	150	0.30	5762	9	250	
19^th^ month	0.02	1.36	2064	15.7		
21^th^ month	0.02	1.28	1772	10		Started
23^th^ month	<0.01		155	12.3		+
25^th^ month	<0.01		169	14.7		+
29^th^ month	<0.01	1.5	165	13.5		+

TSH: thyroid-stimulating hormone; RAI: radioiodine.

**Figure 6:**
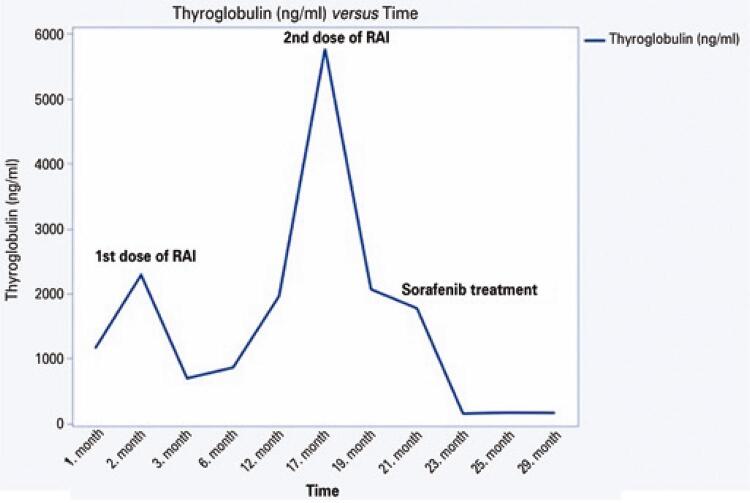
Graph depicting the serum thyroglobulin variation following the initiation of radioiodine and sorafenib treatments after the surgery

Consequently, gamma knife radiation was administered as an additional treatment for the pituitary mass. The patient’s symptoms and bitemporal hemianopsia improved after the surgery and radiotherapy. Although the patient showed clinical improvement, Tg levels increased to 1960ng/mL in 6 months during the most intense period of the COVİD-19 pandemic. FDG-PET/CT revealed increased FDG uptake in the bilateral thyroid bed, right jugular lymph nodes, and lymph nodes lateral to the sternocleidomastoid muscle; decreased uptake in the bilateral lung nodules; progression in the lytic lesion of the left 9^th^ rib; and no uptake in the pituitary gland. As the ultrasonography findings were consistent with the FDG-PET/CT findings, the patient underwent a second neck surgery. Preoperatively, the right central and lateral neck were explored; however, slimy tissues were dissected. Histopathological examination was negative for metastasis or residual disease and revealed granulomatous inflammation and granulation tissue. The pathologist commented that this may have occurred because of the RAI treatment. A second RAI treatment (250 mCi) was planned, and the patient was referred to the Nuclear Medicine Department. However, due to the COVİD-19 pandemic, the patient was treated with a second dose of RAI 5 months after this decision. The basal Tg level decreased from 5762ng/mL to 2064ng/mL 1 month after the second RAI treatment. Based on the Multidisciplinary Council’s decision, sorafenib treatment was deemed appropriate. The patient could not tolerate 800mg of sorafenib daily; therefore, on day 4 of treatment, the dose was reduced to 400mg daily. Since then, the patient has been prescribed 200mg/day. The Tg level was 155.1ng/mL 2 months after the start of treatment and 165.5ng/mL after 6 months of treatment. No serious drug-related adverse effects were observed. Serum thyroglobulin variations after treatment initiation are shown in figure 6. No residual lesions were observed on the last pituitary MRI. The patient is being followed-up at the Endocrinology and Metabolism Clinic and has been stable on replacement treatment.

Written informed consent was obtained from the patient for the publication of this case report and the accompanying images.

## DISCUSSION

Differentiated thyroid cancer has an excellent prognosis, and only approximately 5% of patients have distant metastases, with the most common sites being the lungs and bones.^(10)^ Pituitary metastases are infrequent, although they may be life-threatening and are associated with a worse prognosis. Furthermore, their large size and aggressive behavior lead to mass effects and hypopituitarism. According to a recent review, 30 cases of PM originating from DTC were reported between 1957 and 2018.^(11)^ The diagnosis of PM-induced hypopituitarism in patients with DTC may be delayed because of the absence of specific symptoms, and differentiating PM from other pituitary pathologies using imaging modalities can be difficult.

In the past, diabetes insipidus was reported to be the most common presenting symptom of PM; however, in recent reports, the incidence of visual field defects, cranial nerve palsies, and hypopituitarism has been higher. Pituitary metastases may be the first manifestations of several cancers and can closely mimic symptoms related to pituitary adenoma.^(11)^ Pituitary metastases should be considered in the differential diagnosis of pituitary dysfunction.^(12)^ The current patient presented with symptoms related to hypopituitarism, but did not have diabetes insipidus.

According to a review of 425 patients with PM, thyroid cancer was diagnosed in 13 patients (3.1%).^(7)^ Papillary thyroid cancer (PTC) accounts for 50% of the reported PMs originating from the thyroid, possibly because of its characteristically less aggressive nature.^(12)^

Pituitary metastases management includes surgery, radiosurgery, whole-brain radiotherapy, and chemotherapy. The type of primary malignancy and presence of other metastases determine the prognosis of PM. Survival rates were not affected by surgery alone, with or without radiotherapy. Therefore, palliative measures to decrease morbidity and increase the quality of life should be the aim of management in these patients.^(7)^ Radioiodine-avid metastases from thyroid cancer have better survival rates than those with RAI-refractory metastases.^(10)^ A significant decrease in Tg levels after RAI treatment suggests RAI avidity in these patients. Therefore, a second dose of RAI was administered, after which an increase in the Tg levels was observed.

Pituitary metastasis is generally diagnosed in the advanced stages of malignant disease; however, there have also been cases of sellar masses as the first manifestation of malignancy, as observed in the current patient.^(13)^ In this patient, papillary thyroid cancer was diagnosed before the diagnosis of PM.

Imaging studies may also contribute to the differential diagnosis of sellar masses. Infiltration of the pituitary stalk, cavernous sinus, or posterior lobe; dumbbell-shaped lesions (due to indentation by the diaphragma sellae); rapid tumor growth over time; and bony erosion without sellar enlargement observed on MRI might be important clues for the diagnosis of PMs.^(5,7)^ The MRI of the current patient showed dumbbell-shaped, sellar- and suprasellar-enhancing lesions with cavernous sinus invasion and optic chiasm compression.

Diffuse FVPTC is an uncommon variant of PTC characterized by extensive multinodular involvement of at least one lobe of the thyroid gland. This variant occurs primarily in young females and patients with diffuse FVPTC have substantially increased vascular and lymph node invasion, multicentricity, and extrathyroidal extension compared to those with other types of FVPTC.^(3)^ The current case involved an elderly male patient with no central or lateral lymph node metastasis, although histopathological examination revealed microscopic lymphovascular invasion. The patient had jugular vein thrombosis, suggesting vascular invasion. Increased rates of BRAF V600E mutations have been reported in patients with diffuse FVPTC^(3)^ but the mutation analysis of the current patient did not reveal any common mutations (BRAF, RAS) related to aggressive thyroid cancer. To our knowledge, this is the first reported case of diffuse FVPTC with pituitary metastasis.

This case showed that the presence of hypopituitarism and absence of diabetes insipidus in the initial evaluation of a patient with a pituitary lesion does not exclude the diagnosis of pituitary metastasis. Although distant metastases are rare and usually observed in the lungs and bones, PM should be considered in patients with thyroid cancer, especially in those with aggressive behavior. Pituitary metastases may be the initial manifestation of various cancers or occur during treatment. Unfortunately, the imaging features of pituitary lesions alone are generally insufficient to correctly diagnose PM, which may delay a definitive diagnosis before pituitary surgery. Pituitary dysfunction and visual field defects in patients with a pituitary mass may be indications for neurosurgical intervention, which may guide both diagnosis and treatment. The management of PTC and the role of treatment modalities in prognosis depend on the biological behavior of the tumor. Early diagnosis and multidisciplinary management are crucial for the treatment of these patients.

Sorafenib is a multikinase inhibitor that has received regulatory approval for targeted treatment of progressive radioiodine-refractory differentiated thyroid cancer (RAIRD).^(14)^ According to the decısıon 3 study, overall survival after sorafenib treatment in patients with progressive thyroid cancer was 41.5 months.^(15)^ According to the National Comprehensive Cancer Network/American Thyroid Association consensus on the optimum timing to start TKI therapy in patients with RAIRD, it is recommended that TKI therapy be started in cases of metastatic, rapidly progressive, symptomatic, and/or life-threatening disease in the near future.^(14)^ The current patient had a gradual increase in thyroglobulin levels 2 months after the first dose of RAI. Sorafenib treatment was initiated after the second dose of RAI, after which the thyroglobulin level decreased to approximately 165ng/mL and remained stable for the last 6 months.
